# Adding the Coping Power Programme to parent management training: the cost-effectiveness of stacking interventions for children with disruptive behaviour disorders

**DOI:** 10.1007/s00787-020-01638-w

**Published:** 2020-09-13

**Authors:** Camilla Nystrand, Maria Helander, Pia Enebrink, Inna Feldman, Filipa Sampaio

**Affiliations:** 1 Department of Public Health and Caring Sciences, Uppsala University, Uppsala, Sweden; 2 Department of Clinical Neuroscience, Karolinska Institute, Stockholm, Sweden

**Keywords:** Cost-effectiveness, Cognitive behavioural therapy, Parent management training, Child psychology

## Abstract

Parent management training (PMT) programmes and child cognitive behavioural therapy are recommended approaches for treatment of oppositional defiant disorder in children, and combining these may be effective. However, little is known regarding the economic efficiency of this additive effect. A within-trial cost-effectiveness analysis was carried out in Sweden including 120 children aged 8–12 who screened positive for disruptive behaviour disorders, within a psychiatric care setting, and their parents. They were randomly assigned to either the Swedish group-based PMT Comet, or to an enhanced version, where an additional child component was provided, the Coping Power Programme (CPP). Child behaviour problems as well as healthcare and educational resource use were measured at baseline, post-test and at two-year follow-up. A net benefit regression framework was used to estimate differences in costs and health outcomes between the two intervention arms during the two-year period. Comet with CPP cost on average 820 EURO more per family than Comet only. At the 2-year follow-up, there were 37% recovered cases of ODD in Comet with CPP, in comparison to 26% in the Comet only arm. At a willingness-to-pay of approximately 62,300 EURO per recovered case of ODD, Comet with CPP yielded positive net benefits, in comparison to Comet only. Offering children the CPP simultaneously as their parents receive PMT, in comparison to only providing PMT, yields clinically relevant gains. Despite the relatively small cost for CPP, investment in combining PMT and CPP should be guided by resource prioritisation. Trial registration number: ISRCTN10834473, date of registration: 23/12/2015

## Introduction

Oppositional defiant disorder (ODD) is a disruptive behaviour disorder, characterised by oppositional, argumentative behaviour, angry, irritable mood, or vindictiveness lasting for at least six months [1]. The prevalence of ODD is found to vary between 2 and 14% in epidemiologic samples and 28–50% in clinical samples [2]. ODD is further associated with secondary mood-, anxiety-, impulse control-, and substance use disorders [3]. Elevated ODD symptoms are, in addition, associated with higher levels of conduct problems over time and affected children face a higher probability of committing crimes [4]. ODD also constitutes a risk factor for the development into conduct disorder (CD) [1], and associated antisocial behaviours [5]. Additionally, disruptive behaviour such as ODD and CD are associated with a substantial financial burden [6]. Foster and Jones followed a cohort of children between ages 11 and 18 in the US and estimated total public expenditures due to a CD or ODD, in relation to children without a diagnosis, but from poor neighbourhoods [7]. They found that at the age of 18, the additional costs over the seven year period exceeded $59,000 (in year 2000 dollars) per child with CD, or almost $16,000 for a child with ODD [7]. A majority of the costs accrued to the educational sector (61% of the costs for children with ODD, or in total an additional $10,000 compared to children without a diagnosis). Further, differences increase with age, as costs at the age of 28 for children with CD are estimated to be 10 times higher than for children without a diagnosis [8]. Consequently, avoiding the longer-term consequences related to disruptive behaviour could result in savings for multiple payers. In addition to reducing the individual burden of disease, it is also important from an economic viewpoint to halter the progression of ODD symptomatology by intervening early, especially considering scare public resources that are financing healthcare.

Evidence and recommended practices suggest that parent management training (PMT) programmes as well as child cognitive behaviour therapy (child-CBT) are effective treatments for disruptive disorders, a term summarising both ODD, CD and disruptive behaviour disorder not otherwise specified (NOS) [9, 10]. PMT has shown effective for treatment of disruptive behaviour disorders in numerous meta-analyses [11–14].

In PMT, parents are taught strategies for handling behaviour problems and improving the quality of the parent–child relationship. However, PMT only might not be as effective if the domain of problems a child has does not correspond to difficulties that PMT aims to address [15]. PMTs do not directly target children’s social skills and strategies to deal with anger; thus, there might be a need for targeted interventions for the child as well. Thus, adding a child focused component to PMT, such as child-CBT, has shown added effectiveness [16–18]. In child-CBT, the child is trained in anger management, cognitive problem-solving skills, social skills and perspective taking [16, 19].

The general economic evidence for PMT is widely known, where most evaluations, regardless of targeting externalising problems, or aimed to improve the general health amongst children, have shown to be cost-effective [20]. The costs of PMT vary depending on the type of PMT and country of delivery. In Sweden, the costs range between US$ 700 and 2550 (in 2015 prices) [21].

Two previous economic evaluations have been conducted, stacking interventions such as PMT and teacher training [22], as well as PMT, teacher training and child-CBT [23] for the reduction of ADHD and disruptive behaviour, respectively. Both studies showed cost-effective results in comparison to no intervention. No economic evaluations have been conducted where simultaneously delivered parent and child directed treatments for ODD has been compared to only providing PMT. Further, many previous evaluations lack a societal costing perspective and few use multidimensional outcome measures, which limits comparison across studies and disease areas.

To allocate scarce resources within child and adolescent mental health services (CAMHS), and to build national recommendations for efficiency within these services, economic evaluations are key. In addition to the scarce literature on the topic, most health economic evaluations employ a traditional estimation of incremental costs and health outcomes (which combined provides an estimate of the cost-effectiveness ratio (ICER)) to estimate the differences between groups, in relation to a decision-makers’ willingness-to-pay (WTP). However, it is recognised that this approach is related to methodological shortcomings, such as the true WTP being unknown, especially for clinical outcomes, the statistical uncertainty around the ICER estimate and sub-group variability [24]. The net benefit regression approach has, therefore, been suggested and used as an alternative approach, as it offers solutions to the conventional problems with using ICERs [25, 26]. This approach has yet to be used for economic evaluations of interventions within CAMHS.

In summary, the societal costs for children with disruptive behaviour disorders are substantial, while it is also of great importance to treat these disorders early to prevent an antisocial development. For clinicians and policy-makers, it is valuable that the treatments on offer are effective at reducing disruptive behaviour at a reasonable cost. The cost-effectiveness of PMT has been evaluated previously, while the economic evidence for stacking interventions, such as adding child-CBT to PMT, is lacking. The current paper seeks to employ the net benefit regression technique to estimate the cost-effectiveness of combining PMT and child-CBT, in relation to PMT alone.

## Methods

### Study design

A within-trial economic evaluation was carried out alongside a randomised controlled trial (RCT) with measurements at baseline, post treatment, and at one- and two-year follow-up. An effectiveness evaluation at post treatment has been published previously elsewhere [18]. Children (*n* = 120) aged 8–12 were recruited through Swedish outpatient child and adolescent psychiatric services if they screened positive for oppositional defiant disorder, conduct disorder or disruptive behavioural disorder NOS. Parent-rated baseline data were available for 118 children (Comet = 55 and CPP = 63). Almost three quarters (73%) were boys, and 67% had an ADHD diagnosis [1]. Exclusion criteria were the presence of autism, intellectual disability and severe comorbid psychiatric disorders that required other treatment. Demographic information can be found in Table 1.

**Table 1:** Demographics at baseline for the study population

	Comet + CPP	Comet
	*n* (%)	*n* (%)
Number of participants	*n* = 63	*n* = 55
Male	48 (76.2)	40 (72.7)
Age, mean (SD), in years	9.33 (1.16)	9.36 (1.29)
ADHD diagnosis	43 (68.3)	35 (63.6)
Parent educational level		
Elementary + high school	34 (54.0)	28 (50.9)
University	29 (46.0)	27 (49.1)

*ADHD* attention-deficit/hyperactivity disorder, *CPP* coping power program, *SD* standard deviation

### Interventions

Participants were block randomised at six child and adolescent outpatient psychiatric clinics, to receive PMT or PMT in addition to child-CBT. The PMT evaluated was the Swedish programme Comet [27], inspired by the Incredible Years [28] and PMT-Oregon model [29]. Comet has previously shown to be effective in reducing behaviour problems in populations with increased levels of problematic behaviour [30, 31] in a Swedish setting. This group-based intervention consists of 11 2.5-h sessions with a maximum of six families in each group. It sets out to improve parent–child relationship, minimise negative reinforcement and use praise, rewards and non-punitive consequences to handle problematic behaviours.

The child-CBT consisted of the child component of the Coping Power Programme (CPP), a manual-based group CBT intervention [32]. Treatment components in the Coping Power Programme include anger management training, problem solving skills training, social skills and perspective taking. An adapted Swedish version was used in the current RCT, consisting of the translated 24 1-h sessions manual [33], reshaped into 15 2.5-h sessions (Helander et al. 2018). Language and contextual changes were made to adapt the manual to the Swedish setting. The intervention has shown promising effects when evaluated outside of a Swedish setting [34, 35], and in Sweden when looking at the post-test results from this trial [18].

### Health outcomes

Health outcomes were collected via questionnaires filled in by parents. The primary outcome in the current analysis was the Oppositional/defiant subscale in the parent-rated Disruptive Behaviour Disorder rating scale (DBD-ODD) [36], an 8-item sub-scale with scores ranging between 0 and 3 (not at all to very much). The ODD subscale corresponds to the diagnostic criteria of an ODD diagnosis. The clinically significant reliable change index (CS/RCI) [37] was used to estimate changes on the ODD subscale between baseline and the two-year follow-up. Baseline scores were subtracted from follow-up scores and divided by the standard error of the differences (RCI), where a score between − 1.96 < RCI > 1.96 was considered as a reliable statistical change at a significance level of *p* < 0.05. The CS cut-offs were set at the 95th percentile of problems for boys and girls separately, estimated in the normal population (unpublished data) in Sweden, using method C [37]. Children were considered “recovered” if they had crossed the cut-off (from above to below the cut-off) and done a reliable change (RCI > 1.96) between baseline and the 2-year follow-up. The proportion of “recovered” children was used as the primary outcome in the economic evaluation.

The secondary outcome was quality-adjusted life-years (QALYs), derived from the Strength and Difficulties Questionnaire (SDQ) [38], an instrument with five subscales measuring emotional problems, conduct problems, hyperactivity/inattention, peer problems and prosocial behaviour. To obtain health utilities from the SDQ, a published mapping algorithm was used to predict Child Health Utility 9 dimensions (CHU9D) scores [39]. The CHU9D is a generic preference-based instrument created for children 7–11 years, with items across domains of worry, sadness, pain, sleep, tiredness, annoyance, school, daily routine and activities, each with five response categories. It focuses on the health impact related to quality of life, rather than impairment [40]. Utility valuations based on CHU9D scores and preference weights range between 1.0 (perfect health) and 0.33 (worst state) and were used to estimate QALYs. To map SDQ to CHU9D, an ordinary least squares model was used, with good predictive values of mean group observed utility [41]. Algorithm to map the five SDQ subscales to CHU9D:

$$\mathrm{Utility}= 0.880+\left(- 0.019 \times \mathrm{emotion}\right)+\left(- 0.009 \times \mathrm{conduct}\right)+\left(- 0.001 \times \mathrm{hyperactivity}\right)+\left(- 0.008 \times \mathrm{peer} \mathrm{problems}\right)+\left(0.005 \times \mathrm{prosocial}\right)$$

Individual QALY scores were estimated over a 2-year period: between pre- and post-test (4 months), between post-test and the first-year follow-up, and between first-year and 2-year follow-up using the area under the curve method [41]. These scores were thereafter averaged across intervention arms. The method takes into account both the amount of time and changes in utilities between the different time points.

A 3% discount rate was applied to both DBD-ODD and CHU9D scores between years one and two, as recommended in Sweden [42].

## Resource use and costs

Intervention costs were only estimated for CPP, as Comet was delivered in both conditions. Costs were based on information collected during the trial, and estimations were made for potential resources needed if implemented in clinical practice. Time needed for facilitators (clinical staff including psychologists and social workers) to be trained and supervised, as well as for delivering the intervention (including preparation time), was multiplied by the average hourly salary including social fees and holiday allowance [43]. The same unit costs applied to the staff delivering the training and supervision. Material and venue costs were estimated based on trial data and cost estimations for group sessions at CAMHS [44]. Detailed cost data can be found in Table 2.

**Table 2:** Total intervention cost for the Coping Power Programme (2020 EURO)

Item	Quantity	Total cost facilitator + trainer
**Training cost**		
Training session (time spent by facilitator + trainer)	12 h	418.1
Supervision time needed during the first year (time spent by facilitator + trainer)	8 h	278.7
Booster session (time spent by facilitator + trainer)	2 h	69.7
Number of facilitators + trainer for the study	13 individuals	
Number of facilitators for clinical practice	21 individuals	
Total for the study (quantity × cost)		9964
Total for implementation in clinical practice (quantity × cost)*		16,096
20% of training costs (for implementation in clinical practice)^c^		3219
**Cost of delivery**		**Total cost for the clinic**
Introduction meeting	1 session	46
Number of sessions	15 sessions á 2,5 h	690
Preparation time + time after each session	16 sessions á 2,7 h	1336
Individual meetings	1,75 h per individual	265
Cost of venue	€12 á 16 sessions	193
Cost of materials (per group)		74
Refreshements	6€ á 16 sessions	97
Total per group		2701
Total for all groups in the study (*n* = 14)		37,814
Total for implementation in clinical practice (*n* = 20)^a^		54,020
**Total cost for the study**		
Training + delivery		47,778
Cost/child for the children that started CPP (*n *= 58) (training + delivery)		824
Cost/child for those who participated in 80% (< 12 sessions) of CPP (*n* = 37)		1291
Total cost if implemented in clinical practice		
Training + delivery		70,116
Cost/child		584
20% of training costs + delivery^c^		57,239
Cost/child		477

The amount of children that were randomized and started CPP = 58. Two facilitators are needed per group, with one trainer responsible for all training. The amount of children that would receive the intervention if implemented in clinical practice = 120 (20 facilitators trained per training session, who in total can have 20 groups per year with 6 children in each. The cost for a group session differs from the cost of preparation/time spent after session, as well as the individual meetings, due to differing rates. 2019 price lists for regional services were used

*CPP* Coping Power Program

*For all costs denoted with “implementation in clinical practice”—these are only used as a sensitivity analysis

^a^We assume that training sessions can be held bi-annually—one in the spring semester and one in the autumn, with ten facilitators being trained in each session

^b^Estimated based on the max number of children per group (*n* = 6) and from how many groups can be held by the 20 facilitators yearly (*n* = 20)

^c^If assumed that new facilitators need to be trained every 5 years, hence training costs are spread over 5 years

Costs were collected from a limited societal perspective, including direct costs related to medical use by the children, and resources used at school. Data on resource use were collected at baseline, post-test and at both follow-up assessments, using a questionnaire created specifically for this study. The survey asked binary questions regarding different types of resources used (“Have you ever used any of the following”, with alternatives such as psychologist, counsellor, special education teacher, etc.), followed by “When did you start to use this service?”. The questionnaires were completed by the parents. No recall time was stipulated in the questions asked; hence, if the same resource was used at two consecutive time points, it was assumed that the resource was consumed constantly over that full period of time. In case the resource use began or discontinued at a certain time point, we assumed it to have begun/discontinued right in-between the two measurement time points. At baseline, a recall period of three months was assumed, since it has been suggested as the optimal length [45].

Medication use included consumption related to disruptive behaviour disorders, such as stimulants or antipsychotic drugs. Participants were not asked to provide information regarding the quantity of drugs consumed; hence, it was assumed that each individual took the recommended dose and, therefore, consumed an average amount of drugs per month, as stipulated by the Swedish Association of the Pharmaceutical Industry [46]. Costs for the drugs were estimated by multiplying frequencies by unit costs, using national tariffs of market prices [47]. An average price was estimated based on all packaging forms available.

Services used in school consisted of special education teacher, counsellor, psychologist, classroom assistant or “other”. To estimate costs, both timing, frequency of use and sometimes amount of hours were needed. If no information was given regarding the frequency of resource use at baseline, we assumed the data to be missing. If information regarding frequency of use was missing at other time points, information from baseline were used, if available. For instance, if frequency was not provided at follow-up but provided at baseline, we assumed the same frequency. If no frequency was reported for “classroom assistant” at baseline, one staff working half-time was assumed. A classroom of 20 students was assumed. If “daily support in school” was reported under the category “other”, the presence of one full-time assistant was assumed. The frequency of resource use was multiplied by the respective unit cost, estimated based on average hourly salaries including social fees and holiday allowance, retrieved from Statistics Sweden [43].

Costs were collected in 2018 SEK and then converted to 2020 EURO (€) using a conversion rate based on purchasing power parities [48]. A discount rate of 3% was applied to costs between years one and two. Costs for the resources used at baseline, post-test and two-year follow-up are shown in Table 3.

**Table 3:** Frequency, average use of resources, total cost per participant and health outcomes

Item	Baseline				Post-test				1-year follow-up				2-year follow-up				Incremental difference (Comet + CPP vs. Comet only)
	Comet + CPP		Comet		Comet + CPP		Comet		Comet + CPP		Comet		Comet + CPP		Comet		
	(*n* = 63)		(*n* = 55)		(*n* = 60)		(*n* = 39)		(*n* = 41)		(*n* = 29)		(*n* = 51)		(*n* = 31)		
Average no. of individuals' resource use	n	Mean	n	Mean	n	Mean	n	Mean	n	Mean	n	Mean	n	Mean	n	Mean	
Services at school																	
Special education teacher	11	0.17	12	0.22	13	0.22	9	0.23	5	0.12	3	0.10	14	0.27	5	0.16	–
School counselor	3	0.05	8	0.15	2	0.03	5	0.13	2	0.05	2	0.07	6	0.12	1	0.03	–
School psychologist	2	0.03	5	0.09	1	0.02	1	0.03	0	0.00	0	0.00	2	0.04	1	0.03	–
Classroom assistant	11	0.17	14	0.25	11	0.18	10	0.26	7	0.17	6	0.21	10	0.20	6	0.19	–
Other	10	0.16	5	0.09	11	0.18	6	0.15	6	0.15	6	0.21	9	0.18	3	0.10	–

Average cost per participant	Mean (95% CI)		Mean (95% CI)		Mean (95% CI)		Mean (95% CI)		Mean (95% CI)		Mean (95% CI)		Mean (95% CI)		Mean (95% CI)		2-year
	(*n* = 63)		(*n* = 55)		(*n* = 63)		(*n* = 55)		(*n* = 63)		(*n* = 55)		(*n* = 63)		(*n* = 55)		Mean (95% CI)
Coping Power programme																	
Cost for the trial	823.74		–		–		–		–		–		–		–		–
Services used at school	2,124.1	(641–3,709)	2719.2	(885–4257)	2620.9	(928.5–6386.8)	2486.2	(− 732.6–6599.2)	3801.4	(59–3830)	2468.4	8738.56	(3847–14,475)	5582.05	(189–10,396)		356.5 (− 780.1–9187.0)
Medication	39.2	(− 7.0–92.8)	50.9	(− 14.2–126.2)	43.4	(4.3–245.5)	86.3	(3.3–386.1)	211.1	(55.3–384)	143.7	(− 26.7–333.7)	287.23	(188.9–554.2)	160.01	(56.2–482.3)	116.5 (− 76.0–310.6)
Total societal costs^b^	2988	(1480–4585)	2771.1	(914–4358)	2.664	(− 804.8–6481.3)	2.572	(− 621.2–6875.3)	4012.4	(203.3–7996.3)	2612.1	(− 1297.9–6694)	9.025	(4137.3–14,906)	5,742.06	(344–10,822)	4106.74 (− 748–9383)

Health outcomes	Mean (95% CI)	Mean (95% CI)	Mean (95% CI)		Mean (95% CI)		Mean (95% CI)		Mean (95% CI)		Mean (95% CI)		Mean (95% CI)		
	(*n* = 63)	(*n* = 55)	(*n* = 63)		(*n* = 55)		(*n* = 63)		(*n* = 55)		(*n* = 63)		(*n* = 55)		
Recovered cases of ODD	–	–	25.4%		14.5%		21.6%		16.7%		37.1%		25.8%		OR = 1,70 (0.69–4.1)
QALY^c^	–	–	0.25	(0.24–0.27)	0.25	(0.23–0.27)	0.75	(0.74–0.77)	0.74	(0.73–0.76)	1.49	(1.47–1.50)	1.48	(1.47–1.50)	0.008 (− 0.02–0.04)

*CI* confidence interval, *CPP* coping power program, *DBD* the parent/teacher disruptive behaviour disorder rating scale, *ODD* oppositional defiant disorder, *QALY* quality adjusted life years

Estimates on total cost and health outcomes are based on models using imputed data. All costs are reported in 2020 EURO. Costs at baseline comprise of three months resource use pre-baseline until baseline measurement (we assume a recall period of three months). Costs at post-test are estimated from baseline, hence over 4 months. Costs at the 1-year follow up were estimated from post-test until 1 year (approximately 8 months), and 2-year follow-up is estimated between 1-year follow-up and the two-year time point, hence over approximately 12 months. The incremental cost difference report in the last column comprises of differences between Compet + CPP and Comet at the 2 year follow up. The incremental difference of the Total societal costs include intervention costs

^a^Mean estimates were estimated using generalised linear mixed models (for costs) and linear mixed models (for health outcomes). No adjustments made in the models. 95% confidence intervals were generated by non-parametric bootstraping with 5000 replications

^b^At baseline, this item includes costs for the trial (CPP), services used at school and medication costs. At other follow ups, it includes medication and services used at school

^c^Values correspond to difference in total quality-adjusted life-year (QALY) gains over the trial period, estimated using the area under the curve method

### Statistical analyses

The base-case analysis included all participants with baseline data (*n* = 118), and employed an intention-to-treat approach using multiple chained equations with predictive mean matching to impute missing values [49]. Baseline differences in the DBD-ODD subscale and CHU9D scores were assessed using a *t* test. A logistic model was employed to estimate the probability of being “recovered” at the two-year follow-up, while differences in total QALYs over time were assessed using a linear mixed regression model. Baseline DBD-ODD and utility scores were controlled for in the analyses [50]. Differences in resource use at baseline were assessed using a Wilcoxon rank-sum test, accounting for the non-normality in the distribution of costs. Generalised linear mixed models (GLMM) were used to estimate differences in costs between groups. The results from these analyses are presented in Table 3. Raw data were cleaned and managed in Excel 2016, while all statistical analyses were performed in R Studio V.3.4.2.

### Net benefit regressions to estimate cost-effectiveness

In the base-case analysis, the proportion of “recovered” cases was used as the primary outcome, while QALY change over time was used as a secondary outcome. Estimates of the total accumulated costs, “recovered” cases and QALYs over the two-year follow-up were used to calculate incremental cost-effectiveness ratios (ICER), dividing the average difference in costs between groups (ΔC) by the average difference in effects (Δ*E*).

The base-case analysis used a net benefit regression approach [24], which estimated the expected net monetary benefit on an intervention (NB_CPP_) against a comparator (NB_Comet_). Incrementally, it can be written as:

$$\mathrm{INB}=\mathrm{WTP} \Delta E-\Delta C$$

where the incremental net benefit (INB) is the difference in the mean net benefit for each group. The INB allows for a comparison of costs and effects in the same regression framework, accounting for correlation between outcomes. The INB can thereafter be used as an outcome variable in a multiple linear regression equation:

$${\mathrm{NB}}_{i}= {\theta }_{0}+ {\theta }_{1}\mathrm{CPP}+ {\theta }_{2}\mathrm{Basecost}+ {\theta }_{3}\mathrm{BaseDBDODD}+,\dots ,+ {\varepsilon }_{nb-p}$$

where NB_i_ is the individual net benefit, CPP is the defined treatment group*, *Basecost and BaseDBDODD represent baseline values of costs and effects, and *ε*_nb-p_ is the regression error term. *θ*_1_ is the difference in net benefit for the two groups being compared. The mean net benefit derived from averaging all individual net benefits yields an unbiased estimate of INB, due to costs and effects being included in the same regression. This also allows for calculation of 95% confidence intervals (CI) correctly, as it accounts for the correlation between effects and costs. Different values of WTP for one unit improvement in the health outcome were used to test how sensitive the cost-effectiveness results were. If mean *θ*_1_ > 0, the intervention was deemed cost-effective.

A range of sensitivity analyses were conducted to test the impact of assumptions made in the analysis, and their effect on the results. These analyses included: (1) assuming implementation in a clinical real-life setting, rather than in a trial setting (which affects the intervention costs), (2) analysis of individuals with complete data on the outcomes of interest (*n* = 66), (3) limiting the perspective to a health care payer perspective, which may be more relevant to the decision-maker, and (4) only including cases who completed at least 80% of the sessions (for CPP).

## Results

The amount of ODD problems at baseline did not differ significantly for Comet with CPP compared to Comet only. No significant differences were found for CHU9D health utilities either. The probability of a “recovered” case of ODD was higher in the Comet with CPP group, in comparison to Comet only [odds ratio of 1.70 (95% CI 0.69–4.1)] at the 2-year follow-up. Differences in QALY gains between the two arms were small in magnitude, Comet with CPP showing lower QALY gains [0.0008 (95% CI − 0.02 to 0.04) for Comet only]. The results did not change when controlling for confounders, as seen in Table 4.

**Table 4:** Regression estimates used to estimate the Incremental Net Benefit

Variable	Regression equation	Estimate	Std. error
Cost, Δ*C* (in EURO)	$C\_1 = \beta \_0 + \beta \_1{\text{ CPP}} + \beta \_2{\text{ Basecost}}$	4267.98	2079.2
	$C\_2 = \beta \_0 + \beta \_1{\text{ CPP}} + \beta \_2{\text{ Basecost } + \text{ }}\beta {\text{\_3}}\;{\text{Confounders}}$	4839.07	3133.2
Effect, Δ*D* (2-year RCI "Recovered" cases)*	$D\_{1} = \gamma \_0 + \gamma \_{1}\;{\text{CPP}} + \gamma \_{\text{2 Base DBD}}$	0.54	0.44
	$D\_{2} = \gamma \_0 + \gamma \_{1}\;{\text{CPP}} + \gamma \_{\text{2 Base DBD } + \text{ }}\gamma \_{3}\;{\text{Confounders}}$	0.54	0.45
Effect, Δ*E* (2-year QALY)	$E\_{3} = \alpha \_0 + \alpha \_{1}\;{\text{CPP}} + \alpha \_{\text{2 BaseCHU9D}}$	0.0002	0.01
	$E\_{4} = \alpha \_0 + \alpha \_{1}\;{\text{CPP}} + \alpha \_{\text{2 BaseCHU9D } + \text{ }}\alpha \_{3}\;{\text{Confounders}}$	− 0.0005	0.01
WTP used to estimate ΔNB for primary outcome			
0	${\text{NB}} = \theta \_0 + \theta \_1{\text{ CPP}} + \theta \_2{\text{ Basecost}} + \theta \_4{\text{ BaseDBD}}\_{\text{ODD}} + \theta \_5{\text{ Con founders}} + \varepsilon \_\left( {nb - p} \right)$	− 6382.14	4451.27
10,000		− 5358.59	4632.00
50,000		− 1264.41	6762
62,354		0	7678
70,000		782.68	8275.49
100,000		3853.31	1765.75

Primary outcome–recovered cases of oppositional definat disorder, secondary outcome—QALY. Confounders: age, gender, ADHD diagnosis and parents educational level at baseline

*ΔC* Difference in costs between CPP and CPP + Comet, *ΔD* difference in effects using the DBD-ODD scale, *ΔE* difference in effects using QALYs, *ΔNB* difference in net benefit between CPP and CPP + Comet, *Basecost* baseline value of costs, *BaseCHU9D* baseline value on the CHU9D scale, *BaseDBD* baseline value on the DBD scale, *CHU-9D* child health utility 9 dimensions, *CPP* coping power program, *WTP* willingness-to-pay

*Measured as the probability of a "recovered" case of ODD

Intervention costs for CPP ranged between 824 EURO per child for the trial population, to around 1291 EURO if estimating cost per child for intervention completers (attendance > 80% of the sessions).

Visually, there was no change in resource use over time for the two groups being compared, depicted in the first part in Table 3. Cost of resource use at baseline per participant, estimated over a three-month period before the start of the trial, did not differ significantly between groups, although costs were higher for Comet with CPP arm. The costs varied between 39 [95% CI − 7 to 92.8] and 51 [95% CI − 14.2 to 126.2] EURO for medication use, and service use at school varied between 2124 [95% CI 641–3709] and 2719.2 [95% CI 885–4257] EURO per child for Comet with CPP and Comet, respectively.

The incremental difference in total cost between groups over the trial period did not differ significantly, as shown in Table 3. The total cumulative difference in costs, including medication costs, services used at school and intervention costs, amounted to 4106.7 [95% CI − 748 to 9383] EURO for the two groups, Comet with CPP amounting to higher costs. When controlling for confounders, incremental differences in total costs increased slightly, as depicted in Table 4.

**Fig. 1:**
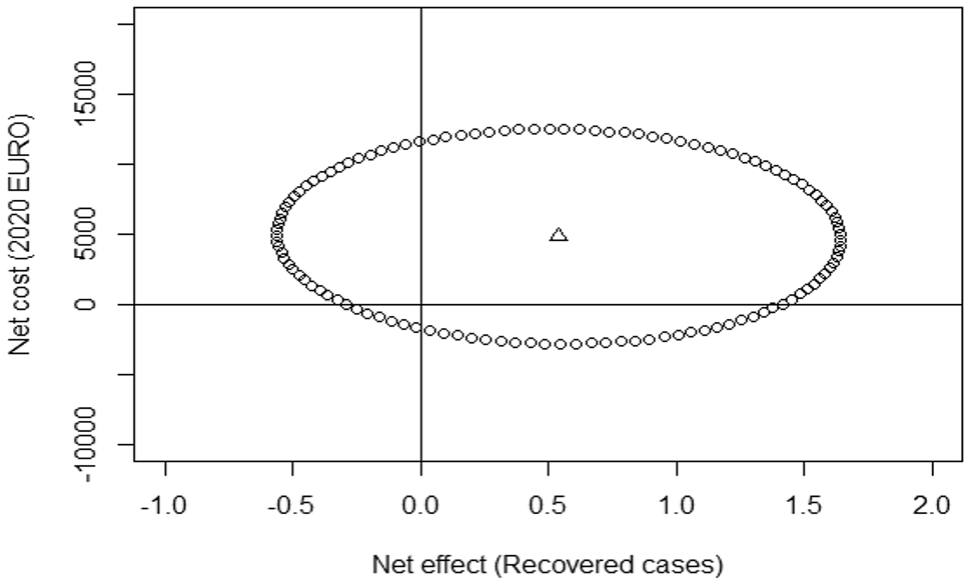
The 95% confidence interval (ellipse) for the adjusted model and the cost and effect estimate from the adjusted models. Δ—Based on the estimates from the cost and effects regressions

Using regression estimates *γ*_1_ and *α*_1t_, the ICER for a “recovered” case of ODD amounted to 8967 EURO, while Comet dominated the Comet with CPP arm with regards to total QALYs gained. Table 4 shows the results from the two-step net-benefit regressions, which was carried out for the primary outcome. The results from the separate regression models for costs and “recovered” cases of ODD adjusted for age, gender, parental education and baseline psychiatric diagnosis, showed slightly changed estimates in comparison to the unadjusted models. The incremental net benefit was estimated using different levels of WTP values. At a WTP value of 62,354 EURO per recovered case, Comet with CPP yielded a positive net benefit (*θ* > 0) in comparison to Comet only, using “recovered” cases as the outcome measure. Figure 1 shows the 95% CI from the adjusted regression model, in addition to the cost and effect estimates.

**Fig. 2:**
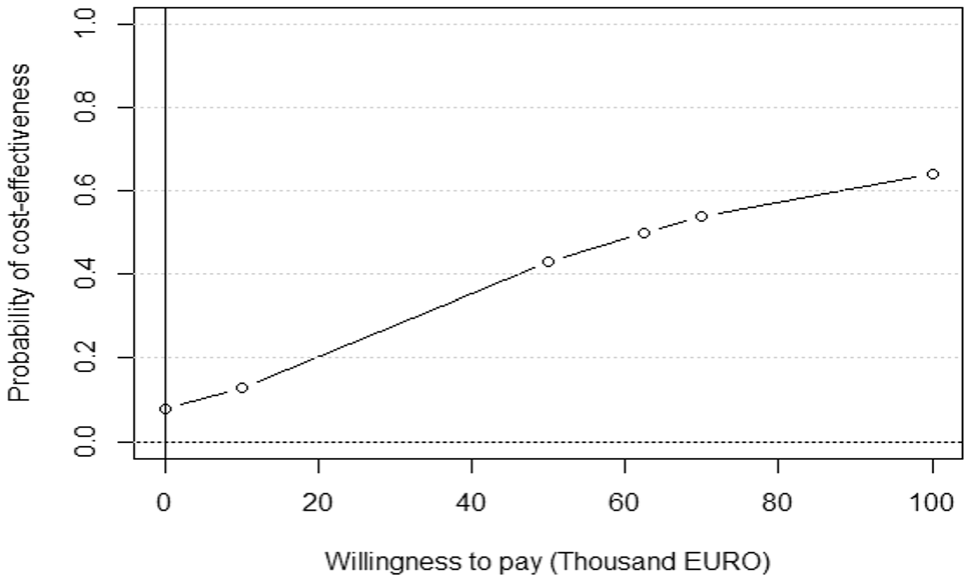
Cost-effectiveness acceptability curve for the base-case analysis. Willingness-to-pay for one recovered case of oppositional defiant disorder (ODD), estimated using the CS/RCI method [38], identified by the disruptive behaviour scale instrument (ODD subscale)

Figure 2 shows the probabilities of cost-effectiveness, illustrated as cost-effectiveness acceptability curves (CEACs) related to different levels of WTP, ranging from zero to 100,000 EURO. The likelihood of cost-effectiveness for Comet with CPP ranged between 7 and 64%, in relation to Comet only, at WTP values of zero to 100,000 EURO, respectively.

**Fig. 3:**
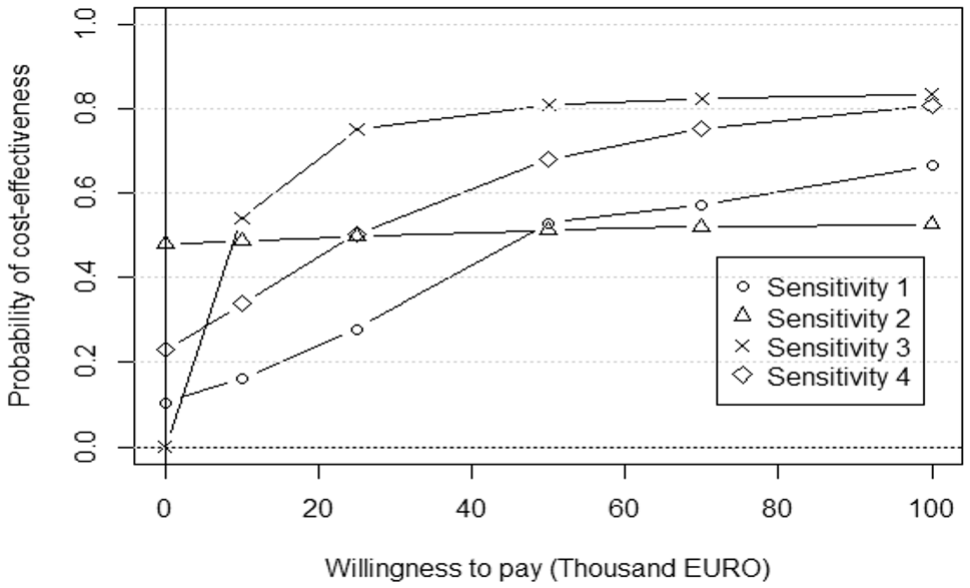
Cost-effectiveness acceptability curves for all sensitivity analyses. Different sensitivity analyses include: (1) implementing the trial in a clinical setting, (2) complete case analysis, (3) health care payer perspective, (4) participation in CPP > 80%

Results from the sensitivity analyses are shown in Fig. 3. The probability of cost-effectiveness at a WTP threshold of 100,000 EURO ranged from 53 to 84% throughout all sensitivity analyses. Using only cases with complete data (*n* = 66) had a large impact on the cost-effectiveness of Comet with CPP, in comparison to Comet only. Regardless of the WTP threshold, the probability did not increase to more than 53%. When including only medication cost, a 50% probability of cost-effectiveness was reached at around 10,000 EURO. The other sensitivity analyses indicated that the results from the base-case analysis were robust to parameter assumptions.

## Discussion

### Study findings and comparison with other literature

This within-trial evaluation aimed to assess the economic efficiency of combined parent and child training, in relation to parent training only, for the reduction of disruptive behaviour in children. The base-case analysis, estimating the proportion of “recovered” cases over the two-year trial period in relation to resource use over the same timeframe, indicated that combining an intervention for children (CPP) with an intervention for parents (Comet) yielded positive net-benefits when the WTP was approximately 62,350 EURO per “recovered” case, in comparison to Comet only. The probability of cost-effectiveness for combining parent and child training ranged between 8% for a WTP of zero, and 64% using a WTP threshold of 100,000 EURO, in relation to parent training only. Sensitivity analyses showed various results, with a large negative impact from only using cases with complete data. Including only medication use had a substantial impact on the results—the WTP needed for the intervention to have a 50% probability of cost-effectiveness was approximately 10,000 EURO. However, since relatively few individuals used medication and the interventions aim to reduce ODD symptoms, which are generally not treated with medication, these results should be interpreted with caution.

For the secondary outcome, QALY gains, there was a small incremental effect difference, and both treatment conditions showed steady levels of health-related quality of life throughout the trial period. One possible explanation for these results may be that only the primary outcome measures, levels of ODD measured on the DBD-ODD subscale, as well as SDQ, were powered for in the study. CHU9D focuses on broader health issues, rather than measuring levels of specific disease impairments, which may have contributed to difficulties in detecting changes when mapping SDQ to CHU9D due to loss of information. The results of the present study, thus, imply that stacking treatments such as child-CBT and PMT may be money well spent compared to PMT only, in terms of clinically and reliable changes of ODD levels. Judgement of efficiency is, however, dependent on the unknown WTP of the decision-maker.

The findings from this study are to some extent in concordance with similar studies in the field. As previously mentioned, no economic evaluations have been conducted where the combination of PMT and child training has been evaluated against PMT or a control group only, for children with disruptive behaviour disorders. Only one full economic evaluation has been conducted where stacking intervention components was beneficial if both parents, teachers and children received training, or teachers and parents concurrently [23].

### Strengths and limitations

This study is the first full economic evaluation of stacking intervention components for children with disruptive behaviour disorders. Importantly, in this study, the time horizon was rather long, which enabled us to capture longer-term impacts of the interventions in the population studied.

A major limitation of the study is that the economic assessment was based on limited information regarding resource use and lacked outcome data from multiple respondents and data collected from a preference-based instrument, which restricted the authors from conducting a more comprehensive analysis. Because interventions may have an impact on other sectors of society in terms of both resource use and effects, such as social services, the voluntary sector and other individuals, it is difficult to know the true value of interventions if we do not know how it has fully affected resource use. In addition, only medication was used to measure health care resource use, underestimating the true impact on health care consumption. As no validated questionnaire was used to collect data on resource use, and information was missing in the survey used, many assumptions regarding frequency and length of use had to be made, whereby results should be interpreted with caution. A large proportion of the data points had to be excluded, whereby the cost estimates are likely to be underestimated.

Regarding the health outcomes, only parental report was used to determine child ODD. Since child behaviour varies greatly between settings [51, 52], assessment of a child should be collected from multiple informants who view the child in different contexts. Only including parental report might, therefore, provide a one-sided picture of the health of the child. Information was gathered from teachers as well. However, the response rate of the teacher reports on disruptive behaviour was unfortunately too low to be included in the analysis. On another note, using a published mapping algorithm to derive CHU9D utilities from the SDQ allowed us to capture the intervention’s effect on different dimensions of quality of life, with excellent predictive ability [39]. However, the utility values for the CHU9D were derived from an Australian population, with an age span of 5–17 years (mean 11.71), which was wider compared to our population. Although the population was from child- and adolescent psychiatric services and the authors stipulated that the values may be useful for other populations as well, caution as to how these estimates may correspond to Swedish utility valuations should be taken. Utility weights for the CHU9D, derived from a Swedish population, are currently not available. Neither are any other validated preference-based instruments available for a child- and adolescent psychiatric population in Sweden.

### Implications to policy and practice

Albeit study limitations, stacking interventions such as child training in the form of CBT with parent training may be clinically effective for children with more ODD symptoms or at a higher risk for antisocial behaviours [18]. Children in the Comet with CPP condition were to a larger degree clinically recovered than those in the Comet condition, and the present study showed positive net benefits at around 62,350 EURO per “recovered” case of ODD, in comparison to delivering parent training only. This amount can be compared to the public costs associated with ODD. Foster and Jones estimated that the additional public costs over a seven-year period for a child with ODD, in comparison to a child without a disorder (but from a high-risk neighbourhood), were approximately 18,000 in 2020 EURO [7], where 40% accrued to the health care sector. Although treatment guidelines for disruptive behaviour disorders recommend PMT as well as child social and cognitive problem-solving training [10, 53], the results from this study do not provide economic evidence for investing in the provision of both to all patients. It is also worth noting that sub-group analyses in the two-year follow-up study showed that children with high number of ODD symptoms at baseline improved to a greater extent in reduced disruptive behaviour in the Comet with CPP group, in comparison to Comet only. However, the opposite results were found for children with low to moderate baseline ODD [54]. In combination with the results from this study, this indicates that precaution must be taken when prioritising resource allocation for the treatment of child ODD, as sub-groups benefit disproportionately and the additional gain from adding CPP to Comet comes at a large cost. Relevant for policy-makers and clinicians might be that since significantly more children with higher levels of ODD at baseline were “recovered cases” at follow-up [54], it might entail that they are better off in handling their daily life, peer relations and school. This may in turn reduce the need for societal services, thus reducing costs. Since not all costs could be captured in this study, the magnitude of these potential cost-offsets remain unknown. To conclude, the proportion of those estimated as “recovered cases” from ODD is larger for children offered child-CBT simultaneously as their parents receive parent management training, in comparison to only providing parents with training. Although costs are relatively small for the child component, investment in delivering both PMT and CPP depends on the willingness-to-pay for such a prioritisation.
